# Acute-on-Chronic Subdural Hematoma Secondary to Falls Due to Alcoholism

**DOI:** 10.7759/cureus.29503

**Published:** 2022-09-23

**Authors:** Aditi Iyer, Morgan Killian, Thor S Stead, Rohan Mangal, Latha Ganti

**Affiliations:** 1 Biology, The Village School, Houston, USA; 2 Emergency Medicine, HCA Florida Ocala Hospital, Ocala, USA; 3 Medicine, The Warren Alpert Medical School of Brown University, Providence, USA; 4 Medicine, University of Miami Miller School of Medicine, Miami, USA; 5 Emergency Medicine, Envision Physician Services, Plantation, USA; 6 Emergency Medicine, University of Central Florida College of Medicine, Orlando, USA

**Keywords:** acute-on-chronic subdural hematoma, alcohol-related head injury, subdural hematoma evacuation, subdural hematoma (sdh), acute subdural hematoma

## Abstract

The authors present a case of a 58-year-old male who is a daily drinker, experiencing bitemporal headaches starting one week prior to seeking medical attention. The patient’s physical examination and vital signs exhibited no irregularities. Imaging studies revealed an acute-on-chronic left subdural hematoma but no intracranial arterial thrombosis or significant stenosis. The patient was managed conservatively due to his intact mental status and did well. The authors discuss alcohol use as a predisposing factor for intracerebral hemorrhage due to the increased risk for head trauma.

## Introduction

A subdural hematoma (SDH) is blood in the space between the dura and the arachnoid mater. An acute subdural hematoma (ASDH) is most often secondary to traumatic brain injury (TBI). The most common causes of TBI include falls and motor vehicle collisions [1]. The estimated mortality of ASDH is between 40 and 60% [2]. Chronic subdural hematoma (CSDH) is one of the most prevalent neurosurgical disorders. Its annual incidence is estimated to be 21/100,000 [3]. Most often, SDH occurs secondary to the tearing of the bridging veins. While the diagnosis of an SDH is fairly straightforward on non-contrast head CT, several mimics are recognized and include cerebral venous thrombosis, dural-based benign masses or neoplasms, epidural hematoma, arachnoid cyst hemorrhage, intracranial hypotension, subdural empyema, and subdural hygroma [4]. An acute-on-chronic is when an “old” collection of blood and blood breakdown products lead to acute subdural bleeding with even minor trauma. Clinically, this often presents with symptoms such as vomiting, slurred speech, headaches, and blurred vision [5]. Acute trauma causes disruption of the bridging veins, leading to acute hemorrhage. Following this, there is a proliferation of mesenchymal elements resulting in angiogenesis and further fibrosis. These serve as a substrate for repeated hemorrhage and expansion of the hematoma [6]. The authors present the case of a patient who presented with an acute-on-chronic SDH.

## Case presentation

A 58-year-old male presented to the emergency department (ED) with the chief complaint of headaches in the bitemporal region that had been occurring for one week, along with diplopia for two days. While the patient denied any surgical history, allergies, or daily medications, he stated that he drank several beers on a daily basis after returning from his place of employment.

Upon ED arrival, the patient was stable. His vital signs were temperature 98.8°, pulse 64, respirations 17 breaths per minute, blood pressure 107/68 mmHg, and oxygen saturation of 99%.

**Figure 1:**
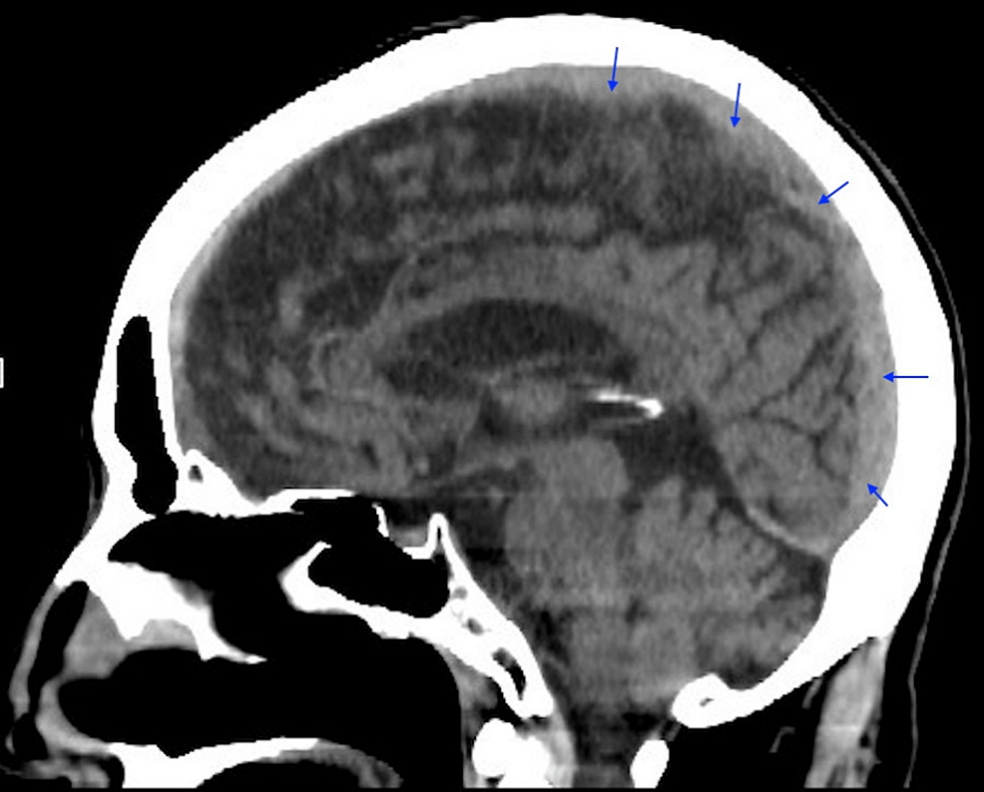
Sagittal view non-contrast brain computed tomography The scan reveals an ill-defined acute-on-chronic left subdural hematoma above the parietal lobe region (arrows).

He had an unremarkable physical examination. On neurologic exam, he had a staggering gait but walked without falling in the ED. He was unable to stand on one foot without losing his balance. His finger-to-nose test, by contrast, was intact bilaterally. His national institutes of health stroke scale (NIHSS) was zero. A non-contrast head CT scan revealed an acute-on-chronic left SDH in the bitemporal region, which measured 0.7 cm in thickness with limited sulcal effacement and no sign of a midline shift (Figure 1).

A subsequent CT angiography demonstrated a minimal acute to subacute component measuring approximately 0.7 cm in thickness. However, no intracranial arterial thrombosis or significant stenosis was observed. The patient was admitted for further workup and neurosurgical consultation.

During his admission to the hospital, the patient’s neurological examination results remained unchanged. The patient underwent magnetic resonance imaging (MRI) that re-demonstrated the medulla oblongata central and left anterolateral subacute hemorrhagic infract, measuring 0.8 x 0.7 x 1.3 cm and the acute left SDH (0.5 cm in width). Given this comprehensive imaging, his stable neurologic status, and a lack of necessity for surgical intervention, the patient was discharged home to follow-up in-clinic. Prior to discharge, the patient was counseled regarding alcohol cessation, as consistent consumption likely resulted in the frequent falls that caused the SDH.

## Discussion

**Figure 2:**
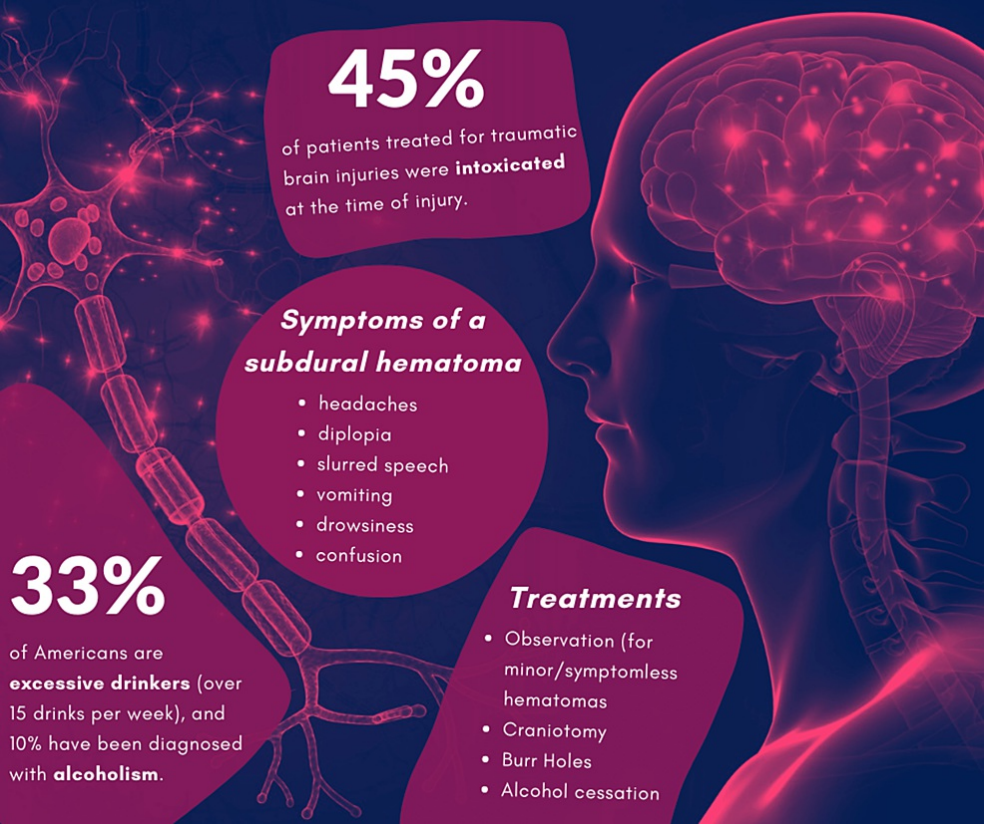
Infographic summarizing alcohol consumption in the United States, alcohol-related brain injuries, symptoms, and treatments for subdural hematomas. Designed by Aditi Iyer, on canva.com

Alcohol is a depressant of the central nervous system (CNS). Because of this, neurons in the brain configure nerve impulses slower. This can lead to problems with coordination, balance, and stability. Since a fall or minor head injury can cause a chronic hematoma to begin acute bleeding, alcoholics are highly prone to this type of neurologic complication [7]. In fact, among patients with fall injuries, 16% are alcohol-related [8]. In 2018, 45% of patients treated for TBI were intoxicated at the time of injury [9] (Figure 2).

A 2008 prospective study of healthy adults between 16 and 60 years who had ground-level falls found a higher incidence of head injuries in those who consumed alcohol [10]. A 2010 systematic review of over 3300 patients demonstrates a dose-response relationship between alcohol and motor vehicle injury death, with an OR of fatal injury of 1.74 (95% CI: 1.43-2.14) for every 0.02% increase in blood alcohol concentration. At 0.08, the legal limit in most countries, the OR was 13.0 (95% CI: 11.1-15.2) [11]. Perhaps even more germane to our patient’s case is the 2021 study of 844 head injury patients that examined the pre-injury alcohol abuse and the incidence of acute SDHs. This study demonstrated that patients with pre-injury alcohol abuse were more likely to have worsening of their intracranial hemorrhage over time and that pre-injury alcohol abuse was an independent predictor of increased death (OR = 2.96, p-value = 0.001) and decreased favorable outcome (OR = 0.46, p-value = 0.001) [12].

The mainstay of treatment for subdural hemorrhage is surgical evacuation, most often via burr hole [13]. Several non-surgical treatment targets have been explored, and include corticosteroids, atorvastatin, tranexamic acid, middle meningeal artery embolization, and angiotensin-converting enzyme inhibitors. Unfortunately, none have shown conclusive benefits to date [14].

The risk of seizures after CSDH is known, but a large systematic review failed to find any significant reduction in the incidence of seizures in patients with CSDH following the administration of antiepileptic drugs [15].

The decision of whether to evacuate the hematoma depends on the clinical presentation of the patient and the extent of the hematoma. Indications for surgical evacuation of hematomas include a decreased Glasgow Coma Scale (GCS), a hematoma thickness greater than 10 mm, or a 5 mm or greater midline shift [16]. In the absence of these criteria, patients can be managed conservatively, as was the case with our patent.

## Conclusions

Binge drinking and alcoholism remain extremely prevalent issues, despite being life-threatening. This case describes a middle-aged man who was a habitual alcohol drinker who experienced an acute-on-chronic SDH most likely due to alcohol-induced head trauma. Fortunately, his hematoma did not result in a depressed level of consciousness and he was able to be managed conservatively.
